# Post-stroke memory impairment among patients with vascular mild cognitive impairment

**DOI:** 10.1186/s12883-014-0244-6

**Published:** 2014-12-20

**Authors:** Soo-Jin Cho, Kyung-Ho Yu, Mi Sun Oh, San Jung, Ju-Hun Lee, Im-Seok Koh, Hee-Joon Bae, Yeonwook Kang, Byung-Chul Lee, the Korean-Vascular Cognitive Impairment Harmonization Standards Study Group

**Affiliations:** Department of Neurology, Dongtan Sacred Heart Hospital, Hallym University College of Medicine, Hwaseong, South Korea; Department of Neurology, Hallym University College of Medicine, Anyang, South Korea; Department of Neurology, National Medical Center, Seoul, South Korea; Department of Neurology, Stroke Center, Seoul National University Bundang Hospital, Sungnam, South Korea; Department of Psychology, Hallym University, Chuncheon, South Korea

## Abstract

**Background:**

The American Stroke Association/American Heart Association recommended the criteria for diagnosis of vascular cognitive impairment and memory impairment (MI) is a feature in the classification of vascular mild cognitive impairment (VaMCI). VaMCI patients with MI may differ in terms of infarct location or demographic features, so we evaluated the clinical characteristics associated with MI in patients with VaMCI.

**Methods:**

A prospective multicenter study enrolled 353 acute ischemic stroke patients who underwent evaluation using the Korean Vascular Cognitive Impairment Harmonization Standard Neuropsychological Protocol at three months after onset. The association between MI and demographic features, stroke risk factors, and infarct location was assessed.

**Results:**

VaMCI was diagnosed in 141 patients, and 58 (41.1%) exhibited MI. Proportions of men and of left side infarcts were higher in VaMCI with MI than those without (75.9 vs. 57.8%, *P* = 0.03, 66.7 vs. 47%, *P* = 0.02). Multiple logistic analyses revealed that male sex (odds ratio [OR] 3.07, 95% confidence interval [95% CI] 1.12-8.42), left-side infarcts (OR 3.14, 95% CI 1.37-7.20), and basal ganglia/internal capsule infarcts (OR 4.53, 95% CI 1.55-13.22) were associated with MI after adjusting other demographic variables, vascular risk factors, and subtypes of stroke.

**Conclusions:**

MI is associated with sex and infarct location in VaMCI patients.

## Background

Vascular mild cognitive impairment (VaMCI) is a subtype of vascular cognitive impairment (VCI) and commonly follows stroke [1,2]. While VaMCI sometimes improves with time, it is associated with an elevated risk of dementia [3,4]. The American Stroke Association/American Heart Association (ASA/AHA) recommended the following criteria for VaMCI: patient exhibits impairment in at least 1 of 4 cognitive domains (memory, executive/activation, visuospatial, language); display normal or mild impairments in daily living activities; imaging results suggest cerebrovascular disease; a temporal relationship exists between the stroke and the cognitive symptoms. Moreover, it is recommended to apply these criteria in the study of VaMCI after stroke [1,5].

Cognitive impairment after stroke is characterized by disturbance of frontal or executive function, however, several previous studies also indicated that clinical stroke causes subsequent poorer performance in multiple cognitive domains including memory [6-8]. Memory impairment (MI) is a prerequisite for a diagnosis of vascular dementia according to DSM-IV criteria and a feature in the classification of VaMCI by ASA/AHA recommendation [1,9]. Verbal memory declines over 3 years after stroke [10] and impairment of verbal memory is associated with progression to dementia or vascular death [4,11]. Memory domain has been considered as a key domain among all cognitive domains, however, little is known about the clinical characteristics of VaMCI with MI.

The prevalence of MI is about 23–55% from a review paper [12] and is 21.4–66.5% from previous studies about VaMCI or VCI without dementia at 3–6 months after stroke [4,6]. Hippocampal atrophy is generally not associated with VCI [13] and the correlation of MI with white matter hyperintensities, infarct location or laterality were reported after stroke, so the mechanisms of MI in VaMCI or VCI may be multifactorial [12]. Interestingly, previous studies showed some difference in age, gender, risk factors, or hippocampal atrophy according to the subtypes of VaMCI or VCI, but these differences were not evaluated in the manner of multivariate analysis [4,14]. Thus, we hypothesize that VaMCI patients with MI may differ in terms of infarct location or demographic features after adjusting the other demographic features, vascular risk factors, and infarct subtypes or locations.

In addition, neuropsychological protocols previously used to assess VCI have varied with respect to examined cognitive domains [2,4,15]. VCI harmonization standards (VCIHS) recommend the evaluation of four specified cognitive domains to conform with ASA/AHA diagnostic guidelines [16]. The Korean VCIHS neuropsychological protocol (K-VCIHS-NP) assesses four cognitive domains and has been validated for use in acute stroke patients [17]. Therefore, the present study uses the K-VCIHS-NP to investigate the associations between MI and the clinical characteristics of VaMCI patients.

## Methods

### Participants

**Figure 1:**
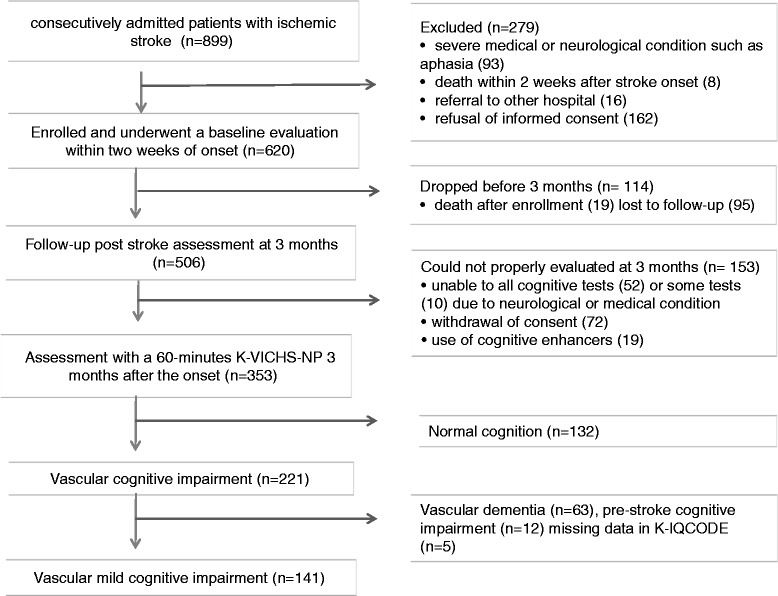
**Patient flow chart.** K-VCIHS-NP, The Korean VCIHS neuropsychological protocol; K-IQCODE, the Korean version of the Informant Questionnaire of Cognitive Decline in the Elderly.

Six hundred twenty acute ischemic stroke patients from 12 hospitals were enrolled in the study within 7 days of symptom onset from October 2007 to August 2008. Exclusion criteria and the exact number of patients for each stage of the study are shown in Figure 1. A detailed description of the methodology has been published elsewhere [17]. Briefly, we conducted a baseline evaluation within two weeks of stroke onset to determine demographic characteristics, vascular risk factors, stroke subtype [18], and functional status. Among these 620 patients, 353 completed the 60-min K-VCIHS-NP evaluation at three months post-stroke.

### Cognitive evaluation

The 60-min K-VCIHS-NP includes 7 tests that assess 4 cognitive domains: the Hopkins Verbal Learning Test for memory; Semantic (animal) and phonemic fluency tests, the Digit Symbol Coding, and the Trail Making Test for executive function/activation; the Rey Complex Figure Test-Copy for visuospatial skills; and the Boston Naming Test for language [19].

We also administered the Korean version of the Informant Questionnaire on Cognitive Decline in the Elderly (K-IQCODE) to examine pre-morbid history of cognitive impairment; the Instrumental Activities of Daily Living scale (IADL) to assess instrumental complex daily functioning; and the Korean version of the Mini-Mental Status Examination (K-MMSE) and Clinical Dementia Rating (CDR) as supplementary tests of cognitive function at 3 months post-stroke. All tests and scales were previously validated for the Korean language and standardized in Korean subjects by age and educational levels [20,21].

### Diagnosis of VaMCI

The diagnostic procedure included a clinical interview and the administration of the K-VCIHS-NP, K-IQCODE, and IADL. Impaired cognitive function was defined as impairment in at least one cognitive domain on the K-VCIHS-NP, as indicated by a score below the 10th percentile using normative corrections for an individual domain. For executive function/activation domains, which consisted of 4 tests, patients who scored below the 10^th^ percentile on more than 2 tests were identified as having impaired functions. Abnormal functional status was indicated by a score greater than 0.43 on the Korean IADL [20]. Pre-stroke cognitive impairment was considered present if the K-IQCODE score exceeded 3.6 [21]. A vascular cause was presumed when cognitive impairment emerged at 3 months after the stroke in the absence of pre-stroke impairment. VaMCI was defined as impairment in at least 1 of 4 cognitive domains, combined with normal or mild impairments in daily living activities and temporal evidence of vascular causation [1].

### Statistical analysis

We compared the demographic features and characteristics of index infarcts of VaMCI patients based on the presence of MI, using a Mann-Whiteny test for continuous variables and chi-square tests for categorical variables. Univariate and multivariate logistic analyses examined the influence of baseline demographic features, infarct locations, and classification of stroke subtypes on memory impairment in VaMCI at 3-month after stroke. We entered age, sex, duration of education, all locations and subtypes of ischemic stroke in the logistic model for detailed description. Analyses were performed with SPSS (Windows version 18.0).

### Ethics statement

This study was approved by the Institutional Review Board/Ethics Committee of each participating hospital (Hallym University Sacred Heart Hospital IRB 2007–137 http://hallym.hallym.or.kr/irb/). All subjects provided informed consents. This study followed Good Clinical Practice guidelines and was consistent with the International Conference on Harmonization of ethical principles for medical research involving human subjects.

## Results

At 3 months post stroke, 506 patients were follow-up and 353 patients completed K-VCI-HP. Cognitive impairments were present in 221 patients, and VaMCI was diagnosed in 141 patients (mean age: 65.5 ± 11.1 years, 92 men and 49 women; Figure 1). Causes of follow-up loss at 3 months were death (n =19) and move away or transfer to another hospitals (n = 95) and the causes of not completing K-VCIHS-NP at 3 months are deteriorated neurological or medical conditions (n =52), withdrawal of consent (n = 72), missing data of cognitive test (n = 10), and use of cognitive enhancers (n = 19). Among the 141 VaMCI patients, MI was present in 58 patients (41.1%). Executive/activation function was most commonly impaired (96, 68.1%), followed by visuospatial function (60, 42.6%), and language function (38, 27%). MI was more frequent in men than women (47.8% vs. 28.6%, *P* = 0.03), while no sex differences were observed in the other domains.

Sixty-nine patients (48.9%) exhibited impairments in two or more domains: There were 17 amnestic single domain VaMCI patients, 41 amnestic multiple domain VaMCI patients, 55 non-amnestic single domain VaMCI patients, and 28 non-amnestic multiple domain VaMCI patients. Combined memory and executive/activation impairments were present in 35 patients, but these were not correlated (r = 0.139, *P* = 0.10). Correlations were observed between language and memory impairments (r = 0.888, *P* = 0.01) and language and visuospatial impairments (r = 0.221, *P* = 0.009).

Baseline demographic features, vascular risk factors, location, laterality, multiplicity, classification of index ischemic stroke are shown in Table 1. Proportions of men and of left side infarcts were higher in VaMCI with MI than those without (75.9 vs. 57.8%, *P* = 0.03, 66.7 vs. 47%, *P* = 0.02). VaMCI with MI tended to have a higher chance of having basal ganglia/internal capsule (36.2 vs.21.7%, *P* = 0.06) or cerebellum (13.8 vs.4.8%, *P* = 0.06) as the location of index stroke than those without. The two groups did not significantly differ in education levels; the vascular risk factors; the other location or the number of infarcts; TOAST classification (Table 1).

**Table 1:** **Comparison of baseline demographic features, vascular risk factors, and location of cerebral infarcts between vascular mild cognitive impairment patients with and without memory impairment**

	**Memory impairment**		***P v*** **alue**
	**Present (n = 58)**	**Absent (n = 83)**	
Age	64.2 ± 9.2	66.4 ± 12.2	0.09
Sex, men (%)	44 (75.9)	48 (57.8)	0.03
Previous stroke (%)	8 (13.8)	8 (9.6)	0.44
Hypertension (%)	35 (60.3)	55 (66.3)	0.47
Diabetes (%)	25 (43.1)	30 (36.1)	0.40
Hyperlipidemia (%)	15 (25.9)	12 (14.5)	0.09
Duration of education (yr)	4.9 ± 1.8	4.7 ± 1.6	0.34
NIH stroke scale score	3.9 ± 3.46	4.5 ± 4.85	0.99
Presense of left infarct (%)	38 (66.7)*	39 (47)	0.02
Location of infarcts			
Cortex (%)	21 (36.2)	39 (47.0)	0.20
Corona radiata (%)	15 (25.9)	27 (32.5)	0.39
Basal ganglia/internal capsule (%)	21 (36.2)	18 (21.7)	0.06
Thalamus (%)	9 (15.5)	6 (7.2)	0.12
Brainstem (%)	7 (12.1)	7 (8.4)	0.48
Cerebellum (%)	8 (13.8)	4 (4.8)	0.06
Multiple infarcts (%)	25 (43.1)	28 (52.8)	0.26
TOAST classification			0.36
Large artery disease	23 (39.7)	38 (45.8)	
Small vessel occlusion	17 (29.3)	18 (21.7)	
Cardioembolim	12 (20.7)	12 (14.5)	
Other or undetermined	6 (10.3)	15 (18.1)	

yr, year; NIH, National Institutes of Health; TOAST, Trial of Org 10172 in Acute Stroke Treatment * one is missing in the information about stoke laterality, so a total of 57 patients with MI was analyzed.

VaMCI with MI had a higher score in sum of box of CDR (2.0 ± 1.9 vs.1.3 ± 1.3, *P =* 0.04) and showed a tendency of lower score in K-MMSE (24.1 ± 5.3 vs. 25.7 ± 3.4, *P =* 0.14) three months after stroke. There are no difference in K-IQCODE (3.0 ± 0.53 vs. 3.0 ± 0.49) and instrumental ADL (0.19 ± 0.37 vs. 0.19 ± 0.32) between two groups.

Logistic analyses with baseline demographic features, vascular risk factors, and location and classification of stroke revealed that male sex (odds ratio [OR] 3.07, 95% confidence interval [95% CI] 1.12-8.42), left-side infarct location (OR 3.14, 95% CI 1.37-7.20), and basal ganglia or internal capsule infarct location (OR 4.53, 95% CI 1.55-13.22) were associated with MI (Table 2). Additional logistic analyses with all above variables and K-IQCODE showed similar results: Male sex (OR 3.48, 95% CI 1.21-10.02), left-side infarct location (OR 3.15, 95% CI 1.37-7.24), and basal ganglia or internal capsule infarct location (OR 4.86, 95% CI 1.64-14.43) were associated with MI, while no such association was observed for other variables (data not shown).

**Table 2:** **Univariate and multivariate analysis for predictor of memory impairment**

	**Univariate**		**Multivariate**	
	**OR (95% CI)**	***P*** **value**	**OR (95% CI)**	***P*** **value**
Age	0.98 (0.95-1.01)	0.25	0.98 (0.93-1.02)	0.27
Men	2.29 (1.09-4.81)	0.03	3.07 (1.12-8.42)	0.03
Education, year	1.07 (0.87-1.31)	0.53	0.83 (0.61.-1.12)	0.22
Previous stroke	1.50 (0.53-4.26)	0.45	2.04 (0.55-7.57)	0.29
Hypertension	0.78 (0.39-1.55)	0.47	1.17 (0.46-2.93)	0.97
Diabetes	1.34 (0.67-2.65)	0.41	1.49(0.65-3.39)	0.34
Hyperlipidemia	2.01 (0.88-4.82)	0.09	1.93 (0.69-5.45)	0.21
Left infarct	2.26 (1.12-4.54)	0.02	3.14 (1.37-7.20)	0.007
TOAST classification				
Large artery disease	0.78 (0.39-1.54)	0.47	0.90 (027–3.03)	0.86
Small vessel occlusion	1.45 (0.69-3.23)	0.30	2.12 (0.53-8.49)	0.29
Cardioembolim	1.54 (0.64-3.73)	0.34	2.20 (0.47-10.60)	0.32
Infarct location				
Cortex	0.64 (0.32-1.27)	0.20	0.97(0.32-2.91)	0.95
Corona radiata	0.72 (0.34-1.53)	0.40	0.71 (0.26-1.92)	0.50
Basal ganglia/internal capsule	2.05 (0.97-4.33)	0.06	4.53 (1.55-13.22)	0.006
Thalamus	2.36 (0.79-7.03)	0.12	2.70 (0.71-10.22)	0.14
Brainstem	1.49 (0.49-4.50)	0.48	1.78 (0.37-8.57)	0.47
Cerebellum	3.16 (0.90-11.05)	0.07	4.05 (0.81-20.14)	0.09

OR, odds ratio; 95% CI, 95% confidence interval; TOAST, Trial of Org 10172 in Acute Stroke Treatment.

## Discussion

The present study showed that, after adjusting for age, sex, stroke risk factors, education levels, and infarct location, VaMCI patients with MI were more likely to be male and have left side, basal ganglia, or internal capsule infarcts. The follow-up rate with K-VCIHS-NP of patients in prospective acute stroke cohort was 69.8% among 506 patients who were followed-up at 3 months after stroke in this study (56.9% among baseline 620 patients) and the proportion of cognitive assessing was somewhat low compared to the previous studies [15,22]. This discrepancy might be explained by the difference in clinical setting as a multicenter setting and in study design excluding those with cognitive enhancer or in extensiveness of the neuropsychological protocol [17].

Sex differences in VaMCI have been indicated by several studies [4,14,23]. For example, males are more likely to have amnestic MCI [4,14], and females recover more quickly from VaMCI [23]. While age-related cognitive decline, vascular risk factors, and stroke are more common in men, it is unclear whether males are more susceptible to cognitive insult by vascular burden or aging [24-26]. In addition, males were at subtle disadvantage when performing this memory test in a previous paper; thus, this should be considered during the rehabilitation or cognitive testing of stroke patients [27].

Infarct location influenced the domain of cognitive impairment. Basal ganglia and thalamic infarcts have been associated with MI in previous studies [7,28-31]. The basal ganglia is known to play a some role in the regulation of memory and the association between MI and basal ganglia infarcts is a line with the previous studies [29,30,32]. MI was related to left-side infarcts and was correlated with language impairment in this study. While verbal memory tasks rely on intact language function, an association between left-side infarct and MI was also found when memory was assessed using non-verbal memory tests [10,33]. Thalamic infarcts were not related to MI in this study. In a study with acute stroke patients, associations between infrequent infarct location and cognitive impairment may be difficult to detect if comparisons are not made with participants who have not suffered a stroke.

The strength of this study is that it investigated the clinical details of VaMCI with MI using ASA/AHA guidelines for VaMCI diagnosis and VCIHS protocols [1,16]. The exclusion of subjects with pre-stroke vascular dementia or cognitive impairment does not guarantee that the included participants did not have Alzheimer’s disease or another form of dementia that contributed to their MI. MI in VaMCI patients in this study may occur from the current ischemic stroke or underlying degenerative disease, but the VaMCI with MI is worthy to evaulate because of high risk of future dementia and death [12,13].

This study has several limitations. First, we did not measure white matter or infarct volume; or Alzheimer’s biomarkers, such as hippocampal volume and apolipoprotein E subtypes [13,14,34]. The study about MI in VaMCI should be adjusted by these variables to confirm the associations. Second, our results were not validated by the long-term VaMCI outcome. Third, we only used the Hopkins Verbal Learning Test to assess memory. While the Hopkins Verbal Learning Test is the only VCIHS-recommended memory test [35,36], adding visual memory or composite score of several memory tests would be informative as an index of MI in stroke patients [30]. Fourth, the wide confidence interval of the association between MI and stroke location may be related to the relatively small number of patients in each location. The association between MI and this parameter should be interpreted with the caution.

## Conclusions

Sex and stroke location differ between VaMCI with MI and those without MI. VaMCI with MI was more frequently men and associated with infarcts on the left side or within the basal ganglia or internal capsule. These differences might be considered in the interpretation of cognitive testing or rehabilitation of VaMCI patients.
